# PseudoGeneQuest – Service for identification of different pseudogene types in the human genome

**DOI:** 10.1186/1471-2105-9-299

**Published:** 2008-07-02

**Authors:** Csaba Ortutay, Mauno Vihinen

**Affiliations:** 1 Institute of Medical Technology, University of Tampere, FI-33014 Tampere, Finland; 2 Research Unit, Tampere University Hospital, FI-33520 Tampere, Finland

## Abstract

**Background:**

Pseudogenes, nonfunctional copies of genes, evolve fast due the lack of evolutionary pressures and thus appear in several different forms. PseudoGeneQuest is an online tool to search the human genome for a given query sequence and to identify different types of pseudogenes as well as novel genes and gene fragments.

**Description:**

The service can detect pseudogenes, that have arisen either by retrotransposition or segmental genome duplication, many of which are not listed in the public pseudogene databases. The service has a user-friendly web interface and uses a powerful computer cluster in order to perform parallel searches and provide relatively fast runtimes despite exhaustive database searches and analyses.

**Conclusion:**

PseudoGeneQuest is a versatile tool for detecting novel pseudogene candidates from the human genome. The service searches human genome sequences for five types of pseudogenes and provides an output that allows easy further analysis of observations. In addition to the result file the system provides visualization of the results linked to Ensembl Genome Browser. PseudoGeneQuest service is freely available.

## Background

Pseudogenes are nonfunctional copies of genes whose transcription or translation is disrupted. They have several biological roles; for example, they may regulate gene expression and create a reservoir of diversity at a genetic or phenotypic level [1]. The recombination between the immunoglobulin V_h _gene and its pseudogenes has been proposed to drive the formation of new genes [2]. Pseudogenes can also drive gene conversion to contribute to immunoglobulin heavy and light chain diversity [3].

Pseudogenes arise either by retrotransposition or duplication of genomic DNA segments [4]. Because of these fundamentally different mechanisms, the different types of pseudogenes have characteristic structures which facilitate identification and classification.

Processed pseudogenes, which emerged via retrotransposons [5], lack introns, a poly A tract at the 3' end, and flanking repeats. Other pseudogenes are complete or partial duplicates of real genes with an interruption in their transcription or translation. The identification of pseudogenes from genomes can be a difficult task. Due to the missing evolutionary pressure, pseudogene sequences evolve neutrally therefore mutations accumulate at a fast pace, i.e. they diverge fast from the original sequences. Homologous sequence stretches missing functional characteristics (such as promoters, interrupted coding regions etc.) are considered pseudogenes.

Two major bioinformatic services have been developed and distributed for the analysis and annotation of pseudogenes. Pseudogene.org [6] is an extensive database and analysis system for pseudogenes from several genomes, including human. The database facilitates the comparison of pseudogenes in selected genomes. The data is collected with an automatic PseudoPipe system [7]. Another service, HOPPSIGEN [8], is dedicated for processed pseudogenes in human and mouse. The database seems to have not been updated recently. Both these services are useful, but have some limitations. If one wants to study recently identified genes then these services are not helpful. The PseudoPipe can be downloaded, but the user should be familiar with programming. As a solution for researchers looking for all types of pseudogenes, as well as for newly identified genes, we developed an online tool that implements the methods for pseudogene identification used in our previous study for tracking human immune system related pseudogenes [9]. At the moment PseudoGeneQuest is the only publicly available web based system, which identifies all types of pseudogenes and pseudogene fragments in the human genome using user provided query sequence as starting point. It is a unique tool for searching and identifying new human pseudogenes.

## Implementation

**Figure 1:**
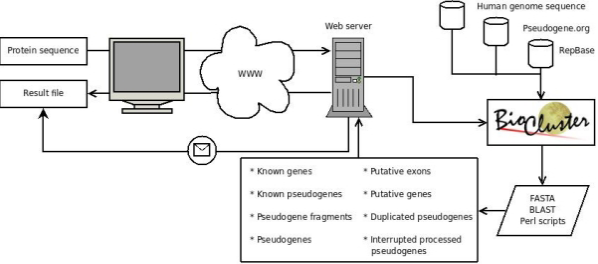
**Layout of the PseudoGeneQuest service**. The user initiates the search by providing a protein sequence on the web page. The analysis is performed on a cluster using databases, search programs and scripts. The results are mailed to the user, and deposited on the web server, where they can be accessed with the provided search ID.

**Figure 2:**
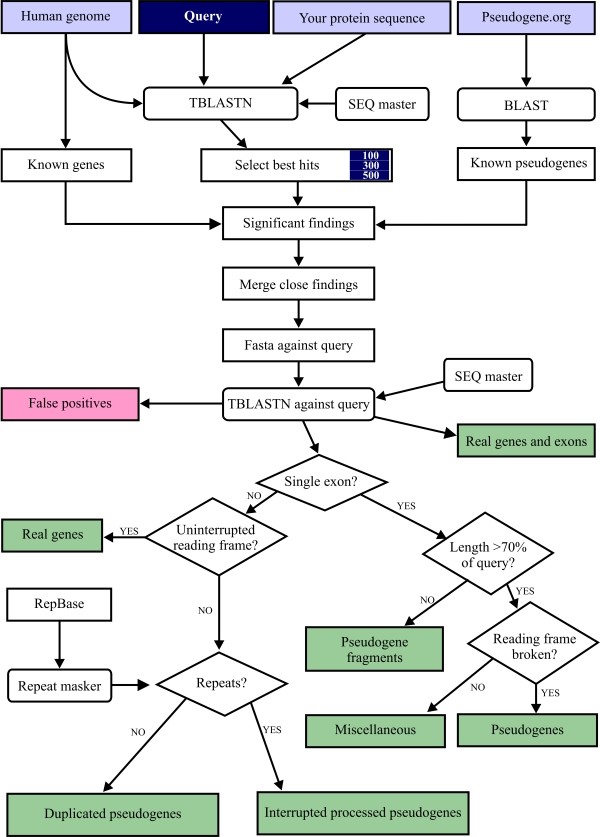
**Overview of the search algorithm of PseudoGeneQuest service**. User provides the protein query sequence and the number of best hits wanted. The algorithm provides seven types of results in addition to the known genes and pseudogenes (see Table 1). The chart is a modified version of Figure 1 from [9].

The components and processes of the system are presented in Figure 1. The search algorithm is a modified version of approach used in Pseudogene.org and published by Gerstein laboratory [10-12] (Figure 2). It was implemented in Perl using Bioperl libraries [13] and runs in the MOSIX2 environment [14]. Briefly, the query sequence is compared using tblastn [15] against the local human genome sequence database. Repeats and low-complexity regions are masked with the SEG masker [15]. The significant findings (with an e-value < 10^-4^) are compared to the data from the human genome and the Pseudogene.org database [16] to exclude already known genes and pseudogenes. The pseudogenes in the Pseudogene.org database are mapped to genome contigs using BLAST.

Hits for the same query that are within 60 nucleotides (nt) of each other are merged as single hits and extended by 30 nt at both ends. These candidates are aligned back to the original query sequence using fastx [17], and the hits with less than 40% amino acid identity are discarded. The pseudogene candidate and the query protein sequences are then aligned using tblastn. If the e-value of the alignment is greater than 10^-10^, the hit is discarded. If the identity is >95%, the length is >95% of the query, and the reading frame does not contain an internal stop codon, the hit is considered a new gene candidate. False positives have an e-value >10^-10 ^or an identity <40% at the amino acid level in the fasta alignment.

Multiexon pseudogene candidates are grouped based on their repeat content as determined by Repeat Masker [18,19]. If the repeat content is <50% of the length of the target, the sequence is identified as a duplicated multiexon pseudogene; otherwise it is classified as an interrupted processed pseudogene.

For pseudogene fragments, the alignment of a single-exon pseudogene candidate covers <70% of the query sequence. If the candidate covers >70% of the known gene, the amino acid identity is >40%, and it contains a stop codon(s) or has a frame shift(s), it is classified as a true pseudogene. In summary, the identified pseudogenes are grouped into five categories: known and newly identified pseudogenes, pseudogene fragments, duplicated pseudogenes, and interrupted processed pseudogenes. In addition, putative new genes and exons can be identified.

The PseudoGeneQuest (PGQ) service runs on a Linux cluster currently consisting of 70 processors. Run times are relatively short; however, since the PGQ is based on an excessive and genome wide analysis, results are not immediately available. Users access the system via a web page [20] and provide either a protein sequence in FASTA format or a protein sequence ID. Each submission has a unique search id, which is used to retrieve results from the web page when ready. The server forwards the job to the cluster, where the actual analysis is performed. The analysis generally takes 5 to 20 minutes. To limit the length of the process we request the user on the query page to specify the maximum number of the hits to be analysed during the process. PGQ offers up 500 hits to analyse, which covers the needs of most users. The user is notified by email about the results.

The results are included in the email, but a more user-friendly version with links is available online. The result file contains hits in each pseudogene category and information for any new genes or gene fragments. The location of each hit in the reference genome sequence is provided. The used human genome build and Pseudogene.org database version is also recorded at the end of the result file together with the version of the PGQ software. The human genome database is updated automatically in our system upon release of new builds.

PGQ groups genome parts aligned to the query into nine categories (Table 1). The result types 'already known genes' and 'known pseudogenes' inform the user about hits which are already available either in the genome annotation or the pseudogene.org database. The type 'real gene or exon' refers to likely coding gene or parts of the gene which codes for the protein provided as query. 'Putative new gene' indicates that the hit is similar to the query and it has uninterrupted reading frame. This might indicate the presence of unannotated exons as was presented in [9], where further analysis of 422 putative new genes found related to human immunome genes revealed two new single exon genes and 36 unannotated exons. 'Pseudogene fragments', true 'pseudogenes' and the 'miscellaneous' results are single exon hits with different coverage of the query sequence and with broken or unbroken reading frames.

**Table 1:** Genome segments identified by PseudoGeneQuest

#	Tag in the result file	Explanation
1	ALREADY KNOWN GENE	Hits overlapping genes annotated in the genome files.
2	KNOWN PSEUDOGENE	Results overlapping with records in pseudogene.org.
3	REAL GENE OR EXON	The hit matches almost exactly to the query. Frequently parts of yet un-annotated or predicted genes.
4	PSEUDOGENE FRAGMENT	Covers <70% of the length of the query. Small parts of pseudogenes.
5	PSEUDOGENE	Covers >70% of the length of the query and the reading frame is broken. Processed pseudogenes with high homology to the query sequence.
6	MISCELLANEOUS	Covers >70% of the length of the query and the reading frame is intact.
7	PUTATIVE NEW GENE	The hit has uninterrupted reading frame. Un-annotated gene.
8	DUPLICATED PSEUDOGENE	Multiexon hit with <50% repeat content. A recent duplication of a gene.
9	INTERRUPTED PROCESSED PSEUDOGENE	Multiexon hit with >50% repeat content. Old processed pseudogenes which accumulated repeats.

'Duplicated' and 'interrupted processed pseudogenes' are multiexon candidates with different repeat content. They might be duplications of real genes or other interrupted pseudogenes. To these sequences different amount of repeats has accumulated after duplication events. Thus, these findings have diverged in a different extent from their ancestor sequences. 'Duplicated pseudogenes' are copies of genomic segment s containing genes with broken gene expression. The duplication happened recently therefore there was not enough time to accumulate repeat sequences. On the other hand, if the duplication took place earlier, the original sequence can be masked by repeat sequences. If more than half of the sequence consist of repeat sequence, then we distinguish it as "interrupted processed pseudogene".

To allow users to link the PGQ results to genomic context, there is a link to connect each of the identified and categorized segments to the genomic contigs in the Entrez database. Further the genome segments be visualized using Ensembl Genome Browser ContigView [21]. This way users can obtain additional information about the identified regions their location in chromosomes.

## Results and Discussion

Originally PGQ was applied for identification of pseudogenes related to 845 human immune system related proteins [9]. The analysis identified a total of 4816 pseudogenes related to 313 genes, along with putative new genes and pseudogene fragments. Most of the new pseudogenes were pseudogene fragments: altogether 3736 fragments for 229 genes. The expression of the pseudogenes varies substantially based on the EST data. Many of these findings were novel and not listed in the Pseudogene.org database. All the results of the earlier extensive analysis are available in the Immunome database [22,23].

The system was also extensively tested with numerous human proteins including cytochrome c protein (NP_061820.1), Bruton agammaglobulinemia tyrosine kinase (NP_000052.1), interferon receptor 2 (NP_000865.2), CD163 (NP_004235.3), tyrosine 3-monooxygenase/tryptophan 5-monooxygenase activation protein zeta (NP_003397.1) and others. The results are shown in Table 2. Most of the hits are known genes or pseudogenes, but all the queries revealed results not available in the databases. Generally those queries, which have more known pseudogenes than known genes, detect several pseudogene fragments (IFNAR2 and YWHAZ). Putative new genes are rarely identified, which is not surprising, since annotation of the human genome improves from build to build.

**Table 2:** Results of test analysis.

**Gene**	**IFNAR2**	**CD163**	**YWHAZ**	**BTK**	**CYCS**
Sequence ID	NP_000865.2	NP_004235.3	NP_003397.1	NP_000052.1	NP_061820.1

Already known gene	15	5	11	32	6
Known pseudogene	35	29	42	27	54
Real gene or exon	18	24	16	36	15
Pseudogene fragment	36	0	9	8	5
Pseudogene	0	0	0	0	0
Miscellaneous	0	2	6	0	5
Putative new gene	0	0	0	0	0
Duplicated pseudogene	2	0	0	0	0
Interrupted processed pseudogene	0	3	0	2	0

Selected genes were tested with PGQ service. The 100 hits with the highest blast score were analysed.

For researchers interested in long list of putative pseudogenes or pseudogene fragments related to a human gene or protein not listed in Pseudogene.org, there is another tool, PseudoPipe [7] available from the authors of the pseudogene.org database. The system is powerful but possibly out of reach for some users if they do not have the necessary skills to install and run the program package. For them and all others, PGQ provides an alternative, which is easy to use and allows extensive analysis of the query sequence. Its results can be compared to the Pseudogene.org database, however can provide additional information because the query sequece provided by the user can be any protein sequence, not just those used for the construction of the Pseudogene.org database. The system yields results, which the users can study further with other experimental or *in silico *methods.

## Conclusion

PGQ is an online tool for users who want to identify pseudogenes related to a query sequence. PGQ groups results in altogether 9 categories (Table 1). The system has a user friendly web interface for placing the queries, uses a powerful computer cluster to handle parallel searches and notifies the user via email about finished analysis. Results are provided to the email and additionally via the PGQ web page, which provides also links to the identified sequences.

## Availability and requirements

PseudoGeneQuest service is available freely for the scientific community via . Non-academic usage is permitted upon agreement with the authors. The users need in addition to the query sequence only a recent recent version of a web browser.

## Authors' contributions

CO implemented the service in the perl. MV designed and coordinated the project. All authors drafted the manuscript and approved its content.
